# Functional Brain Networks in Preschool Children With Autism Spectrum Disorders

**DOI:** 10.3389/fpsyt.2022.896388

**Published:** 2022-07-04

**Authors:** Bin Qin, Longlun Wang, Jinhua Cai, Tingyu Li, Yun Zhang

**Affiliations:** ^1^Department of Radiology, National Clinical Research Center for Child Health and Disorders, Ministry of Education Key Laboratory of Child Development and Disorders, Chongqing Key Laboratory of Translational Medical Research in Cognitive Development and Learning and Memory Disorders, Children’s Hospital of Chongqing Medical University, Chongqing, China; ^2^Children Nutrition Research Center, Children’s Hospital of Chongqing Medical University, Chongqing, China; ^3^Chongqing Engineering Research Center for Clinical Big Data and Drug Evaluation, Medical Data Science, Academy of Chongqing Medical University, Chongqing, China

**Keywords:** preschool children, ASD, rs-fMRI, graph theory, functional connectivity

## Abstract

**Objective:**

The present study aims to investigate the functional brain network characteristics of preschool children with autism spectrum disorder (ASD) through functional connectivity (FC) calculations using resting-state functional MRI (rs-fMRI) and graph theory analysis to better understand the pathogenesis of ASD and provide imaging evidence for the early assessment of this condition.

**Methods:**

A prospective study of preschool children including 32 with ASD (ASD group) and 22 healthy controls (HC)group was conducted in which all subjects underwent rs-fMRI scans, and then the differences in FC between the two groups was calculated, followed by graph-theoretic analysis to obtain the FC properties of the network.

**Results:**

In the calculation of FC, compared with the children in the HC group, significant increases or decreases in subnetwork connectivity was found in the ASD group. There were 25 groups of subnetworks with enhanced FC, of which the medial prefrontal and posterior cingulate gyrus and angular gyrus were all important components of the default mode network (DMN). There were 11 groups of subnetworks with weakened FC, including the hippocampus, parahippocampal gyrus, superior frontal gyrus, inferior temporal gyrus, precuneus, amygdala, and perirhinal cortex, with the hippocampus and parahippocampal gyrus predominating. In the network properties determined by graph theory, the clustering coefficient and local efficiency of the functional network was increased in the ASD group; specifically, compared with those in the HC group, nodes in the left subinsular frontal gyrus and the right middle temporal gyrus had increased efficiency, and nodes in the left perisylvian cortex, the left lingual gyrus, and the right hippocampus had decreased efficiency.

**Conclusion:**

Alterations in functional brain networks are evident in preschool children with ASD and can be detected with sleep rs-fMRI, which is important for understanding the pathogenesis of ASD and assessing this condition early.

## Introduction

Patients with autism spectrum disorder (ASD) are characterized by impaired social interactions and communication skills and typically demonstrate restricted interests and/or repetitive behaviors (1). The prevalence of ASD is increasing yearly, with the CDC reporting a prevalence of 1.7% (2, 3) in children aged 8 years in 2014 versus 1.85% in 2020 (1). The causes of ASD are complex, and it is now recognized that interactions between genetic and environmental factors may lead to early brain changes that result in the development of ASD (4). The diagnosis of autism in children is currently based on typical clinical presentations and an assessment of clinical scales, but some objective indicators and markers are still lacking.

With advances in imaging technology, resting-state functional magnetic resonance imaging (rs-fMRI), based on blood oxygen level-dependent (BOLD) imaging, has been widely used to study functional brain networks in patients with ASD. The form that functional brain connectivity takes in ASD is controversial, with differing views on whether it is under- or overconnected (5), some studies proposing that both types of connectivity abnormalities coexist in the form of reduced long-distance connectivity and increased short-distance connectivity (6), while other studies present evidence to the contrary (7). Uddin et al. reviewed several papers and found that the above inconsistencies may be correlated with patient age, i.e., functional brain connectivity is reduced in adolescents and adults with ASD but elevated in children with ASD (8).

Resting state magnetic resonance imaging imaging generally requires the subject to be awake, so it is often performed on adolescents or adults with ASD who are more compliant than children (9), for whom rs-fMRI is thus difficult to perform. Some researchers (10) have compared functional brain connectivity between awake and sedated states and found that although certain brain regions showed reduced activity after sedation, functional connectivity (FC) was still more significant, suggesting that stable spontaneous brain activity remains under sedation.

In light of these studies, we performed rs-fMRI imaging on children with ASD aged 2–6 years and explored their functional brain network characteristics using FC and graph theory-based brain network analysis methods in the hope of providing an imaging basis for the early assessment of ASD.

## Materials and Methods

### Ethical Approval

This study was approved by the Research Ethics Committee of Children’s Hospital of Chongqing Medical University [IRB number: (2018) Ethical Review (Research) No. (82)]. All parents of the subjects gave written informed consent after they had been informed of the possible risks and benefits of the research and were assured of the security and privacy concerning the children’s medical records.

### Participants

All participants were recruited at the child health care department of Children’s Hospital of Chongqing Medical University, a tertiary hospital and National Children’s Medical Center in China, from July 2014 to July 2016. ASD was rigorously diagnosed according to the DSM-5 criteria (11) and a Childhood Autism Rating Scale (CARS) score of more than 30 and confirmed by the Autism Diagnostic Observation Schedule (ADOS-2). ASD patients were included if they were 2–6 years old and were first-time patients who had not undergone appropriate rehabilitation. Exclusion criteria included other neurological disorders and genetic conditions, such as ASD with fragile X chromosome and Rett’s syndrome. The healthy controls (HC) group was composed of healthy children aged 2–6 years. HC group children were volunteers openly recruited from July 2014 to July 2016 who underwent a general physical examination, MRI routine sequence scan, and the Gesell Developmental Scale (GDS) test. Inclusion criteria included no significant abnormalities in growth and development on the general physical examination, no significant organic diseases or delayed myelination on the MRI routine sequence scan, and GDS score of > 75 in all categories.

### MRI Data Acquisition

Each subject’s MR data were obtained by a 3 Tesla MR scanner (Achieva, Philips, Netherlands) with an 8-channel head coil. Since most of the children under the age of 6 needed to be sedated, all subjects were to undergo sleep deprivation for a certain period of time (usually 6–8 h). Oral 10% chloral hydrate 0.3 ml/kg was administered with a maximum dose ≤ 15 ml. After the subject had fallen asleep, the level of consciousness was tested, and if there was no response to mild painful stimuli, the subject was then placed flat on the examination bed (in the supine position). During the examination, the subject was removed from the study if he or she awoke and was unable to continue with the examination.

Each subject underwent a routine MRI scan first, followed by sagittal three-dimensional SPGR sequencing (3D-SPGR) and rs-fMRI if no significant intracranial lesions were found.

The parameters of the routine MRI sequences were as follows: axial T1WI: inversion recovery sequence (IR), repetition time (TR) = 2,000 ms, and echo time (TE) = 20 ms; axial T2WI: turbo field echo sequence (TFE), TR = 3,500 ms, and TE = 80 ms; T2FLAIR sequence: TR = 8,000 ms, and TE = 125 ms. All axial scanning fields of view (FOVs): 230 mm × 191 mm × 143 mm, slice thickness, 5 mm, interval, 1 mm, and a total of 20 layers.

The parameters of the 3D-SPGR sequence were as follows: TR = 7.7 ms; TE = 3.8 ms; flip angle, 8°; FOV, 256 × 256; voxel size, 1 mm × 1 mm; matrix: 250 × 250; slice thickness, 1 mm; and total time: 155 s.

The parameters of the rs-fMRI were as follows: TR = 2,000 ms, TE = 35 ms, FOV: 192 × 192; matrix: 64 × 64; flip angle, 90°; slice thickness, 4 mm; gap, 0.5 mm; and total time: 8 min 6 s, with a total of 240 time points acquired.

### MR Image Processing and Analysis

Preprocessing of the rs-fMRI data was performed in the SPM12 (SPM Neurology, London, United Kingdom) toolkit. In this study, three patients with HC and one with ASD did not meet the requirements and were excluded. We also calculated the FD (framewise displacement) for each subject, and a two-sample *t*-test revealed no significant differences in FD values between the two groups (ASD mean = 0.175, HC mean = 0.169, *p* = 0.83). The corrected images were normalized to the EPI template, and voxels were resampled to 3 mm × 3 mm × 3 mm.

**FIGURE 1:**
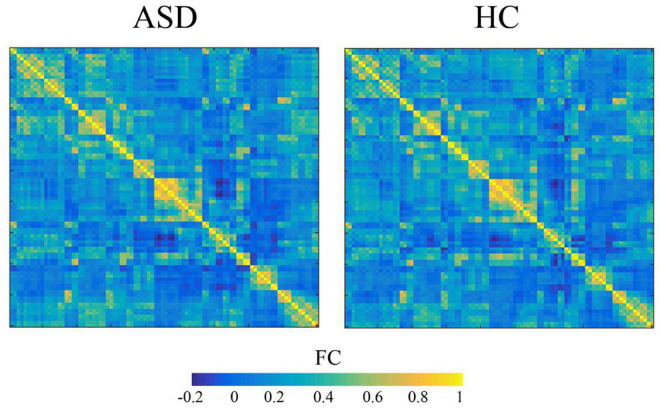
Average functional connection matrix of the ASD group and HC group.

The normalized images were further processed, including spatial smoothing, detrend, filtering, and regression of 24 head movement parameters, the cerebrospinal fluid mean signal and the white matter mean signal. The Anatomical Automatic Labeling (AAL) template cortex and 90 subcortical brain regions were used as regions of interest (ROIs). Anatomical Automatic Labeling (AAL) is a software package and digital atlas of the human brain. It is typically used in functional neuroimaging-based research to obtain neuroanatomical labels for the locations in 3-dimensional space where the measurements of some aspect of brain function were captured. In other words, it projects the divisions in the brain atlas onto brain-shaped volumes of functional data (12). The time series signal of each ROI was extracted, and the Pearson correlation coefficient between the ROIs was analyzed to obtain the correlation matrix for each subject. The Fisher Z-transformation was applied to the correlation matrix to obtain the Z matrix. The values of the Z matrix satisfied a normal distribution and were used for statistical analysis. The average connection matrix for the two groups is shown in Figure 1.

For the ASD and HC groups, a one-sample *t*-test was used to detect those edges where the FC value was significantly not equal to zero. A statistical significance threshold of *p* < 0.05 was assumed, and edges above the threshold formed a connected mask. Comparisons of FC between the two groups were made within this mask. It should be noted that only the positively connected edges were considered for this study value, as the negatively connected edges were not well interpreted in clinical practice.

Network-based Statistic (NBS) was used to test for differences between FC groups. The processing steps for NBS are as follows: Briefly, (1) first test the difference in FC between ASD and control on each edge to obtain the statistical value of each edge; (2) take a statistical threshold of |T| = 3.26 (corresponding to *P* = 0.001), remove edges below the threshold and retain those above the threshold; (3) estimate the connectivity components composed of the retained edges, each of which can be considered as a connectivity sub-network; (4) estimating the significance level of each connected component using a permutation test (5,000 times). Specifically, for each permutation, all subjects were randomly assigned to the ASD and HC groups, and the group differences of each edge were recalculated to obtain the statistical value of each edge, and then the size of the maximum connected component (number of edges) was calculated by retaining the edges above the threshold. After 5000 permutations, the permutation distribution of the maximum connected component was obtained, and the significance level of each true connected component was determined based on this distribution; (5) *P* < 0.05 for the connected component was considered significant.

### Calculation of Network Properties for Functional Connectivity

**FIGURE 2:**
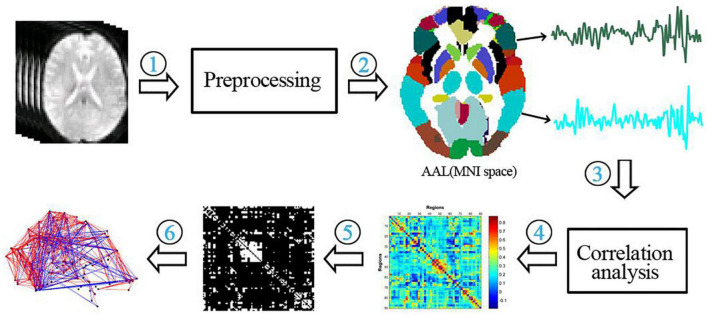
Flow chart of the functional network analysis. (1) preprocessing of functional data; (2) extraction of temporal signals from 90 brain regions of the AAL template; (3) correlation analysis of temporal signals between brain regions; (4) construction of a functional connectivity matrix; (5) thresholding of the functional connectivity matrix; (6) calculation of network properties.

Each subject’s functional network properties were calculated according to its FC matrix. The specific process is shown in Figure 2.

Global properties of the brain networks were calculated, including the clustering coefficient (C), local efficiency (Elocal), shortest path length (L), global efficiency (Eglobal) (L), normalized clustering coefficient (C), normalized shortest path length (L), global efficiency (Eglobal), normalized clustering coefficient (L), normalized shortest path length (L), and small-worldness (S).

### Statistical Analysis

Statistical analysis was performed by using Statistical Package for Social Sciences (IBM-SPSS) software. For each network attribute, we calculated the area under the curve (AUC). A two-sample *t*-test was used to detect differences in network attributes between the ASD and HC groups. For the network global attributes, *P* < 0.05 was considered significant for differences. For the node attributes of the network, the significance threshold range was set at *P* < 1/N (where N is the number of nodes, 90).

## Results

### Demographic Data

The demographic data of the participants are shown in Table 1. Among participants in the ASD group, the chronological age and CARS score were 3.03 ± 0.99 years and 33.47 ± 1.67, respectively. Six of the ASD patients (19%) were female, and 26 (81%) were male. A total of 22 healthy controls were included in the HC group. Among them, 5 individuals (23%) were female, 17 individuals (77%) were male, and the chronological age was 2.95 ± 0.90 years.

**TABLE 1:** Demographic characteristics of the participants.

Variables	ASD (*n* = 32)	HC (*n* = 22)	*P*-value
Age (mean ± SD)	3.03 ± 0.99	2.95 ± 0.90	0.82^a^
Sex (male/female)	26/6	17/5	0.71^b^
CARS score (mean ± SD)	33.47 ± 1.67		

*^a^Two-sample t-test, ^b^Chi-square test.*

### Analysis of Functional Connectivity

**FIGURE 3:**
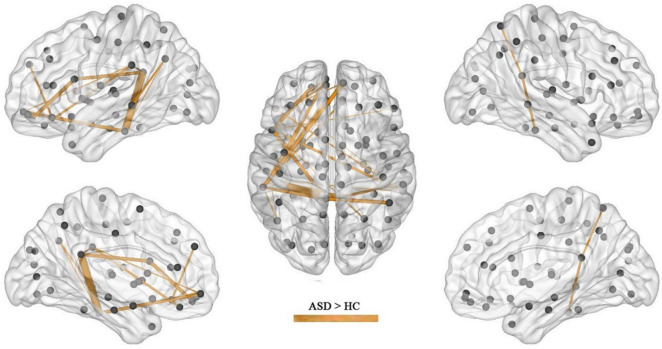
Subnetworks with increased FC in ASD (*p* = 0.003).

**FIGURE 4:**
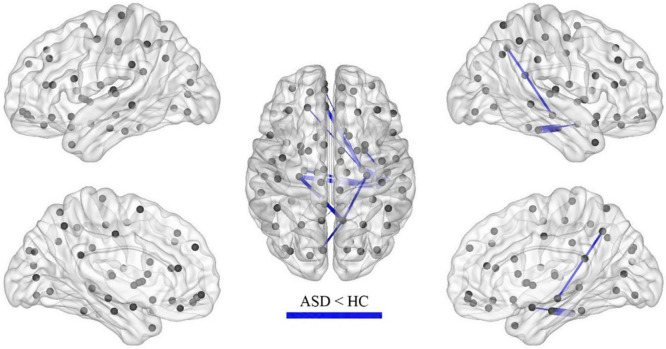
Subnetworks with decreased FC in ASD (*p* = 0.003).

After the calculation of FC, significant increases or decreases in subnetwork connectivity was found, as shown in Tables 2, 3 and Figures 3, 4.

**TABLE 2:** Subnetworks with increased FC in the ASD group relative to the HC group (*p* = 0.003).

ROI i	ROI j	Mean FC in ASD	Mean FC in HC	*T*-value
Frontal_Inf_Oper_L	Frontal_Sup_Orb_L	0.34	0.16	3.84
Temporal_Mid_R	Frontal_Mid_L	0.26	0.08	3.27
Frontal_Inf_Tri_R	Frontal_Inf_Oper_L	0.58	0.39	3.32
Cingulum_Post_L	Frontal_Inf_Oper_L	0.21	–0.02	3.78
Cingulum_Post_R	Frontal_Inf_Oper_L	0.14	–0.05	3.30
Temporal_Mid_R	Frontal_Inf_Oper_L	0.22	0.02	3.65
Temporal_Mid_R	Frontal_Inf_Tri_L	0.28	0.09	3.42
Frontal_Sup_Medial_L	Frontal_Inf_Orb_L	0.61	0.41	3.53
Frontal_Sup_Medial_R	Frontal_Inf_Orb_L	0.48	0.29	3.36
Frontal_Med_Orb_L	Frontal_Inf_Orb_L	0.57	0.35	3.45
SupraMarginal_L	Frontal_Sup_Medial_R	0.16	–0.03	3.90
Temporal_Inf_L	Frontal_Med_Orb_L	0.43	0.20	3.78
Temporal_Inf_L	Frontal_Med_Orb_R	0.33	0.14	3.35
SupraMarginal_L	Rectus_L	0.11	–0.06	3.58
Parietal_Inf_R	Cingulum_Post_L	0.11	–0.12	3.86
SupraMarginal_L	Cingulum_Post_L	0.16	–0.03	3.42
Temporal_Mid_L	Cingulum_Post_L	0.5	0.27	3.80
Temporal_Inf_L	Cingulum_Post_L	0.51	0.21	4.51
Temporal_Inf_R	Cingulum_Post_L	0.45	0.24	3.27
Parietal_Sup_R	Cingulum_Post_R	0.11	–0.06	3.51
Temporal_Inf_L	Cingulum_Post_R	0.37	0.13	3.85
Temporal_Inf_R	Cingulum_Post_R	0.41	0.22	3.60
Temporal_Mid_L	Angular_L	0.58	0.32	3.47
Temporal_Inf_L	Angular_L	0.66	0.40	3.31
Temporal_Inf_L	Temporal_Mid_R	0.58	0.37	3.40

**TABLE 3:** Subnetworks with decreased FC in the ASD group relative to the HC group (*p* = 0.003).

ROI i	ROI j	Mean FC in ASD	Mean FC in HC	*T*-value
ParaHippocampal_R	Frontal_Sup_L	0.05	0.24	–3.37
ParaHippocampal_R	Frontal_Sup_Medial_L	0.15	0.31	–3.29
ParaHippocampal_R	Rectus_L	0.18	0.36	–3.71
Precuneus_R	Hippocampus_L	0.06	0.25	–3.69
Temporal_Inf_R	Hippocampus_L	0.09	0.27	–3.81
Precuneus_L	Hippocampus_R	0.05	0.21	–3.26
Precuneus_R	Hippocampus_R	0.06	0.24	–3.53
Temporal_Inf_R	ParaHippocampal_L	0.09	0.3	–3.55
Temporal_Inf_R	ParaHippocampal_R	0.17	0.41	–4.27
Temporal_Inf_R	Amygdala_R	0.11	0.26	–3.41
Precuneus_R	Calcarine_L	0.36	0.54	–3.49

### Analysis of Brain Network FCs

**FIGURE 5:**
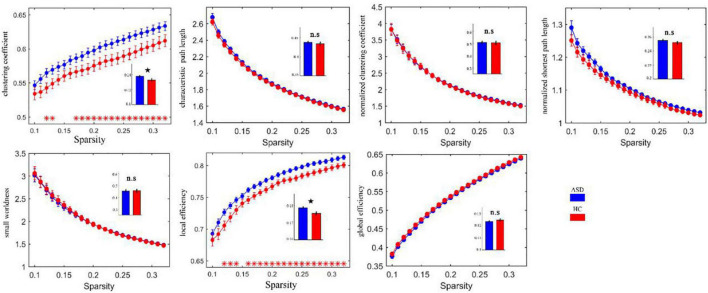
Clustering coefficients and local efficacy of the abnormally elevated network FC in ASD. Red * indicates that the network attributes of the two groups are significantly different under this threshold (*p* < 0.05). The histogram represents the area under the curves of the two groups. The black star indicates that there are significant differences in the AUC values of the two groups’ network attributes. ns, no significance.

Calculating the brain network FC properties through graph theory revealed that the FC network of ASD had small-world properties. It is well known that Watts and Strogatz (13) combined the concept of path length (the minimum number of edges required to establish a connection between nodes) with the concept of clustering coefficients between nodes (i.e., the probability that nodes j and k that are directly connected to node I are also directly connected to each other) to create the concept of small-world properties. This means that the brain networks under study between regular and random networks have both higher clustering properties similar to regular networks and shorter shortest path lengths similar to random networks (14). The stronger the “small world” property of the network. The clustering coefficient (C) and local efficiency (Elocal) were both increased in the ASD group relative to the HC group, as shown in Figure 5.

**FIGURE 6:**
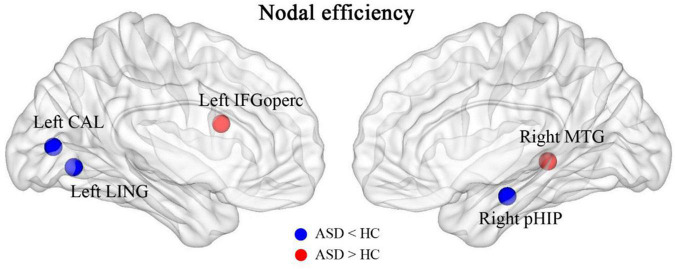
Brain regions with abnormally altered nodal efficiency in ASD. The red brain regions had significantly increased nodal efficiency in ASD. The blue brain regions had significantly decreased nodal efficiency in ASD.

After calculating the nodal efficiency, it was found that the nodes that had increased efficiency in the ASD group were the inferior frontal gyrus, opercular part (left IFGoperc), and middle temporal gyrus (right MTG), and the nodes that had decreased efficiency in the HC group were the left lingual gyrus (left LING), calcarine fissure and surrounding cortex (left CAL), and right parahippocampal gyrus (right pHIP), as shown in Figure 6.

## Discussion

To date, the etiology of ASD remains uncertain. In the past, it had been suggested that ASD is primarily an abnormality in the structure of a particular brain region. More studies have found widespread cortical underconnectivity, local overconnectivity, and mixed results suggesting disrupted brain connectivity as a potential neural signature of autism (15, 16). Resting-state FC has enabled the identification of the primary networks of the brain, and the default mode network (DMN) has been implicated in social-cognitive deficits in ASD (17). Some studies have found that abnormal FC extensively exists within some networks in children with ASD (18), while others have also concluded that children with ASD have decreased FC in a number of regions in the action observation network (19). A diffusion tensor imaging (DTI) study found increased topological network efficiency in preschoolers with autism (20). In a review of numerous studies, Padmanabhan et al. found that the DMN is in a state of increased connectivity during childhood and shows decreasing connectivity with age (21), and Harikumar et al. concluded that the DMN in ASD shows a mixed pattern of strong and weak FC patterns in the DMN. One reason for the mixed trend in ASD could be that different brain regions of the social cognitive network of children with ASD are at different levels of development, such as underdevelopment of specific brain regions of the social cognitive network and reduced connectivity in social cognition-related areas, while other behavioral and connectivity patterns show typical development (17).

The present study found that the brain regions of the DMN were in a state of increased connectivity in preschool children with ASD, which may be related to their symptoms of social impairment. The brain regions with increased FC in the ASD group included the superior frontal gyrus, middle frontal gyrus, inferior frontal gyrus, superior temporal gyrus, middle temporal gyrus, inferior temporal gyrus, posterior cingulate gyrus, superior limbic gyrus, and angular gyrus, of which the medial prefrontal, posterior cingulate, and angular gyrus are all important components of the DMN, an important brain system that processes information about the “self” and the “outside world” and is strongly activated in the absence of external stimuli, while the attention network is activated once external stimuli are perceived, after which the DMN is suppressed.

In this study, in addition to brain areas belonging to the DMN, there was also increased FC in the temporal lobe, including the superior temporal gyrus, middle temporal gyrus and inferior temporal gyrus, areas belonging to the auditory network and responsible for speech perception. A behavioral experiment found that people with early ASD have impairments in speech recognition (22). In addition, people with ASD have difficulty recognizing and learning unfamiliar sounds but are indistinguishable from the general population in recognizing well-known sounds (23). This phenomenon may be related to abnormal brain connections in speech perception (24). The abnormalities in FC in the temporal lobe found in this study may suggest that preschool children with ASD also have abnormalities in speech perception.

The current study also found that FC was decreased in some brain regions of children with ASD, including the hippocampus, parahippocampal gyrus, superior frontal gyrus, inferior temporal gyrus, precuneus, amygdala, and perirhinal cortex of the talar fissure, with the hippocampus and parahippocampal gyrus predominating. The hippocampus and amygdala are key components of the nervous system for emotion regulation and perception regulation, which have been suggested to be associated with the development of ASD in previous studies (25). The decreased connectivity of this brain region found in this study may be associated with emotional alterations in ASD. The perisylvian cortex is part of the emergent network and the present study suggests that FC of the emergent network is reduced in preschool children with ASD, consistent with an enhanced DMN.

Lateralization of brain function or structure can occur in the developing brain. The centers of left-sided brain lateralization include the DMN (posterior cingulate cortex, medial prefrontal cortex, temporal junction) and language areas (e.g., Broca’s and Wernicke’s areas), whereas the centers of right-sided lateralization include the attentional control network (e.g., medial parietal sulcus, anterior insula) (26). Abnormal lateralization of the structure and function of the brain is often associated with neuropsychiatric disorders such as ASD (27, 28). In our study, we also found a tendency for left-sided lateralization in regions of hyperconnected brain networks in children with ASD, which may be related to a tendency for right-sided lateralization of reduced brain network connectivity.

We also found that brain networks in both the ASD and HC groups had small-world properties. The clustering coefficient (C) and local efficiency (Elocal) were both greater in the ASD group than in the HC group, suggesting an increase in both direct and indirect connectivity of nodes in the functionally connected brain network but not in the efficiency of the global network, which is partially consistent with the theory proposed by Belmonte et al. that network performance in ASD patients is characterized by an increase in short-range connectivity and a decrease in long-range connectivity (6). In the ASD group, the nodal efficiency of the FC was enhanced in the left subinsular frontal gyrus and the right middle temporal gyrus and reduced in the left lingual gyrus, the left perirhinal cortex of the talar fissure and the right parahippocampal gyrus relative to the HC group. It is suggested that these brain regions may play a central nodal role in school-aged patients with ASD, but this role should be explored in subsequent studies.

This study still has some limitations and due to the poor completion of natural sleep MRI in younger children, chloral hydrate sedation was chosen to assist in the completion of MRI. A recent study on brain activity in rats pointed out that brain activity in rats sedated with chloral hydrate has a rhythm similar to natural sleep, but of different duration (29). Therefore, our results on FC in the preschool children are not a true rs-MRI and more preparation will be needed to achieve a true resting state MRI, such as more sleep deprivation, late night examination time, more attempts, etc.

## Conclusion

Alterations in functional brain networks are evident in preschool children with ASD and can be detected with sleep rs-fMRI, which is important for understanding the pathogenesis of ASD and assessing this condition early.

## Data Availability Statement

The original contributions presented in this study are included in the article/supplementary material, further inquiries can be directed to the corresponding author.

## Ethics Statement

The studies involving human participants were reviewed and approved by the Research Ethics Committee of Children’s Hospital of Chongqing Medical University [IRB number: (2018) Ethical Review (Research) No. (82)]. Written informed consent to participate in this study was provided by the participants’ legal guardian/next of kin.

## Author Contributions

YZ: designing the topic, collecting data, and writing the manuscript. BQ: MR parameter setting, post-processing, and statistical analysis. LW: scanning and image quality assessment of MR. JC and TL: quality control of the experimental process and the analysis and discussion of the results. All authors contributed to the article and approved the submitted version.

## Conflict of Interest

The authors declare that the research was conducted in the absence of any commercial or financial relationships that could be construed as a potential conflict of interest.

## Publisher’s Note

All claims expressed in this article are solely those of the authors and do not necessarily represent those of their affiliated organizations, or those of the publisher, the editors and the reviewers. Any product that may be evaluated in this article, or claim that may be made by its manufacturer, is not guaranteed or endorsed by the publisher.

## Abbreviations

ASD: autism spectrum disorder
HC: healthy control
rs-fMRI: resting state magnetic resonance imaging
BOLD: blood oxygen level-dependent
ADOS: autism diagnostic observation schedule
CARS: childhood autism rating scale
3D-SPGR: sagittal three-dimensional SPGR sequencing
FD: framewise displacement
ROI: regions of interest
FC: functional connectivity
DMN: default mode network
AAL: Anatomical Automatic Labeling
NBS: Network-based Statistic.

## References

[1] Maenner MJ Shaw KA Baio J Washington A Patrick M DiRienzo M Prevalence of autism spectrum disorder among children aged 8 years – autism and developmental disabilities monitoring network, 11 sites, United States, 2016. *MMWR Surveill Summ.* (2020) 69:1–12. 10.15585/mmwr.ss6904a1 32214087 PMC7119644

[2] Baio J Wiggins L Christensen DL Maenner MJ Daniels J Warren Z Prevalence of autism spectrum disorder among children aged 8 years – autism and developmental disabilities monitoring network, 11 sites, United States, 2014. *MMWR Surveill Summ.* (2018) 67:1–23. 10.15585/mmwr.ss6706a1 29701730 PMC5919599

[3] Christensen DL Braun KVN Baio J Bilder D Charles J Constantino JN Prevalence and characteristics of autism spectrum disorder among children aged 8 years – autism and developmental disabilities monitoring network, 11 sites, United States, 2012. *MMWR Surveill Summ.* (2018) 65:1–23. 10.15585/mmwr.ss6513a1 30439868 PMC6237390

[4] Ziats MN Grosvenor LP Rennert OM. Functional genomics of human brain development and implications for autism spectrum disorders. *Transl Psychiatry.* (2015) 5:e665. 10.1038/tp.2015.153 26506051 PMC4930130

[5] Mash LE Reiter MA Linke AC Townsend J Müller RA. Multimodal approaches to functional connectivity in autism spectrum disorders: an integrative perspective. *Dev Neurobiol.* (2018) 78:456–73. 10.1002/dneu.22570 29266810 PMC5897150

[6] Belmonte MK Allen G Beckel-Mitchener A Boulanger LM Carper RA Webb SJ. Autism and abnormal development of brain connectivity. *J Neurosci.* (2004) 24:9228–31. 10.1523/JNEUROSCI.3340-04.2004 15496656 PMC6730085

[7] Tomasi D Volkow ND. Reduced local and increased long-range functional connectivity of the thalamus in autism spectrum disorder. *Cereb Cortex.* (2019) 29:573–85. 10.1093/cercor/bhx340 29300843 PMC6319176

[8] Uddin LQ Supekar K Menon V. Reconceptualizing functional brain connectivity in autism from a developmental perspective. *Front Hum Neurosci.* (2013) 7:458. 10.3389/fnhum.2013.00458 23966925 PMC3735986

[9] Ha S Sohn IJ Kim N Sim HJ Cheon KA. Characteristics of brains in autism spectrum disorder: structure, function and connectivity across the lifespan. *Exp Neurobiol.* (2015) 24:273–84. 10.5607/en.2015.24.4.273 26713076 PMC4688328

[10] Greicius MD Kiviniemi V Tervonen O Vainionpää V Alahuhta S Reiss AL Persistent default-mode network connectivity during light sedation. *Hum Brain Mapp.* (2008) 29:839–47. 10.1002/hbm.20537 18219620 PMC2580760

[11] Wakefield JC. Diagnostic issues and controversies in DSM-5: return of the false positives problem. *Annu Rev Clin Psychol.* (2016) 12:105–32. 10.1146/annurev-clinpsy-032814-112800 26772207

[12] Tzourio-Mazoyer N Landeau B Papathanassiou D Crivello F Etard O Delcroix N Automated anatomical labeling of activations in SPM using a macroscopic anatomical parcellation of the MNI MRI single-subject brain. *Neuroimage.* (2002) 15:273–89. 10.1006/nimg.2001.0978 11771995

[13] Watts DJ Strogatz SH. Collective dynamics of ‘small-world’ networks. *Nature.* (1998) 393:440–2. 10.1038/30918 9623998

[14] Humphries MD Gurney K Prescott TJ. The brainstem reticular formation is a small-world, not scale-free, network. *Proc Biol Sci.* (2006) 273:503–11. 10.1098/rspb.2005.3354 16615219 PMC1560205

[15] Maximo JO Cadena EJ Kana RK. The implications of brain connectivity in the neuropsychology of autism. *Neuropsychol Rev.* (2014) 24:16–31. 10.1007/s11065-014-9250-0 24496901 PMC4059500

[16] Yao Z Hu B Xie Y Zheng F Liu G Chen X Resting-state time-varying analysis reveals aberrant variations of functional connectivity in autism. *Front Hum Neurosci.* (2016) 10:463. 10.3389/fnhum.2016.00463 27695408 PMC5025431

[17] Harikumar A Evans DW Dougherty CC Carpenter KLH Michael AM. A review of the default mode network in autism spectrum disorders and attention deficit hyperactivity disorder. *Brain Connect.* (2021) 11:253–63. 10.1089/brain.2020.0865 33403915 PMC8112713

[18] Xu S Li M Yang C Fang X Ye M Wei L Altered functional connectivity in children with low-function autism spectrum disorders. *Front Neurosci.* (2019) 13:806. 10.3389/fnins.2019.00806 31427923 PMC6688725

[19] Delbruck E Yang M Yassine A Grossman ED. Functional connectivity in ASD: atypical pathways in brain networks supporting action observation and joint attention. *Brain Res.* (2019) 1706:157–65. 10.1016/j.brainres.2018.10.029 30392771

[20] Qin B Wang L Zhang Y Cai J Chen J Li T. Enhanced topological network efficiency in preschool autism spectrum disorder: a diffusion tensor imaging study. *Front Psychiatry.* (2018) 9:278. 10.3389/fpsyt.2018.00278 29997534 PMC6030375

[21] Padmanabhan A Lynch CJ Schaer M Menon V. The default mode network in autism. *Biol Psychiatry Cogn Neurosci Neuroimaging.* (2017) 2:476–86. 10.1016/j.bpsc.2017.04.004 29034353 PMC5635856

[22] Boucher J Lewis V Collis G. Familiar face and voice matching and recognition in children with autism. *J Child Psychol Psychiatry.* (1998) 39:171–81. 9669230

[23] Schelinski S Riedel P von Kriegstein K. Visual abilities are important for auditory-only speech recognition: evidence from autism spectrum disorder. *Neuropsychologia.* (2014) 65:1–11. 10.1016/j.neuropsychologia.2014.09.031 25283605

[24] Schelinski S Borowiak K von Kriegstein K. Temporal voice areas exist in autism spectrum disorder but are dysfunctional for voice identity recognition. *Soc Cogn Affect Neurosci.* (2016) 11:1812–22. 10.1093/scan/nsw089 27369067 PMC5091681

[25] Groen W Teluij M Buitelaar J Tendolkar I. Amygdala and hippocampus enlargement during adolescence in autism. *J Am Acad Child Adolesc Psychiatry.* (2010) 49:552–60. 10.1016/j.jaac.2009.12.023 20494265

[26] Nielsen JA Zielinski BA Ferguson MA Lainhart JE Anderson JS. An evaluation of the left-brain vs. right-brain hypothesis with resting state functional connectivity magnetic resonance imaging. *PLoS One.* (2013) 8:e71275. 10.1371/journal.pone.0071275 23967180 PMC3743825

[27] Fletcher PT Whitaker RT Tao R DuBray MB Froehlich A Ravichandran C Microstructural connectivity of the arcuate fasciculus in adolescents with high-functioning autism. *Neuroimage.* (2010) 51:1117–25. 10.1016/j.neuroimage.2010.01.083 20132894 PMC2966943

[28] Lange N Dubray MB Lee JE Froimowitz MP Froehlich A Adluru N Atypical diffusion tensor hemispheric asymmetry in autism. *Autism Res.* (2010) 3:350–8. 10.1002/aur.162 21182212 PMC3215255

[29] Ward-Flanagan R Lo AS Clement EA Dickson CT. A comparison of brain-state dynamics across common anesthetic agents in male Sprague-Dawley rats. *Int J Mol Sci.* (2022) 23:3608. 10.3390/ijms23073608 35408973 PMC8998244

